# Combined effects of single nucleotide polymorphisms *TP53 *R72P and *MDM2 *SNP309, and p53 expression on survival of breast cancer patients

**DOI:** 10.1186/bcr2460

**Published:** 2009-12-18

**Authors:** Marjanka K Schmidt, Johanna Tommiska, Annegien Broeks, Flora E van Leeuwen, Laura J Van't Veer, Paul DP Pharoah, Douglas F Easton, Mitul Shah, Manjeet Humphreys, Thilo Dörk, Scarlett A Reincke, Rainer Fagerholm, Carl Blomqvist, Heli Nevanlinna

**Affiliations:** 1 Departments of Epidemiology, Experimental Therapy and Pathology, Netherlands Cancer Institute, The Netherlands, Plesmanlaan 121, 1066 CX Amsterdam, The Netherlands; 2 Department of Obstetrics and Gynecology, Helsinki University Central Hospital (HUCH), Haartmaninkatu 8, 00290 Helsinki, Finland; 3 Strangeway's Research Laboratory, Worts Causeway, CB1 8RN Cambridge, UK; 4 Hannover Medical School, Departments of Gynaecology and Radiation Oncology, Carl-Neuberg-Straße 1, 30625 Groß-Buchholz, Hannover, Germany; 5 Department of Oncology, Helsinki University Central Hospital (HUCH), Haartmaninkatu 8, 00290 Helsinki, Finland

## Abstract

**Introduction:**

Somatic inactivation of the *TP53 *gene in breast tumors is a marker for poor outcome, and breast cancer outcome might also be affected by germ-line variation in the *TP53 *gene or its regulators. We investigated the effects of the germ-line single nucleotide polymorphisms *TP53 *R72P (215G>C) and *MDM2 *SNP309 (-410T>G), and p53 protein expression in breast tumors on survival.

**Methods:**

We pooled data from four breast cancer cohorts within the Breast Cancer Association Consortium for which both *TP53 *R72P and *MDM2 *SNP309 were genotyped and follow-up was available (n = 3,749). Overall and breast cancer-specific survival analyses were performed using Kaplan-Meier analysis and multivariate Cox's proportional hazards regression models.

**Results:**

Survival of patients did not differ by carriership of either germ-line variant, R72P (215G>C) or SNP309 (-410G>T) alone. Immunohistochemical p53 staining of the tumor was available for two cohorts (n = 1,109 patients). Survival was worse in patients with p53-positive tumors (n = 301) compared to patients with p53-negative tumors (n = 808); breast cancer-specific survival: HR 1.6 (95% CI 1.2 to 2.1), *P *= 0.001. Within the patient group with p53-negative tumors, *TP53 *rare homozygous (CC) carriers had a worse survival than G-allele (GG/GC) carriers; actuarial breast cancer-specific survival 71% versus 80%, *P *= 0.07; HR 1.8 (1.1 to 3.1), *P *= 0.03. We also found a differential effect of combinations of the two germ-line variants on overall survival; homozygous carriers of the G-allele in *MDM2 *had worse survival only within the group of *TP53 *C-allele carriers; actuarial overall survival (GG versus TT/TG) 64% versus 75%, *P *= 0.001; HR (GG versus TT) 1.5 (1.1 to 2.0), *P *= 0.01. We found no evidence for a differential effect of *MDM2 *SNP309 by p53 protein expression on survival.

**Conclusions:**

The *TP53 *R72P variant may be an independent predictor for survival of patients with p53-negative tumors. The combined effect of *TP53 *R72P and *MDM2 *SNP309 on survival is in line with our a priori biologically-supported hypothesis, that is, the role of enhanced DNA repair function of the *TP53 *Pro-variant, combined with increased expression of the Mdm2 protein, and thus overall attenuation of the p53 pathway in the tumor cells.

## Introduction

Breast cancer outcome may be affected by germ-line variants in genes that play a role in DNA damage control and repair such as *TP53 *(R72P) and *MDM2 *(SNP309) [1,2]. The Mdm2 protein is a negative regulator of the tumor suppressor protein p53 [3]. The R72P (215G>C) polymorphism of the *TP53 *gene is located in a proline-rich region of p53 suggested to be required for the growth suppression activity of p53 [4] and for its ability to induce apoptosis [5]. The two variant protein forms, R72 (arginine) and 72P (proline), have been shown to differ in their biological functions: the R72 variant is a stronger and faster inducer of apoptosis than the 72P variant [6,7]. The 72P variant also binds more efficiently to iASPP, an inhibitor of pro-apoptotic function of p53, which may be another reason for the inferiority in apoptosis induction of this variant [8]. The 72P variant has been found to be more efficient in inducing cell-cycle arrest [7] and DNA repair [9] than the R72 variant which may protect tumor from chemotherapy-induced apoptosis.

Previous studies have shown that the R72P polymorphism is not associated with increased breast cancer risk [1,10,11]. However, an association of R72P with breast cancer survival has been suggested, though with inconsistent results and possibly only in patients with p53-negative tumors [10-16]. It has also been suggested that patients with the Pro/Pro genotype are less sensitive to anthracycline-based treatment than those with the Arg/Pro or Arg/Arg genotype [14,16], in line with the Pro-allele being more efficient in cell-cycle arrest [7] and DNA repair [9] induction.

A common single nucleotide polymorphism in the *MDM2 *promoter region, a T to G change at nucleotide 309 in the first intron (-410G>T; named SNP309), has been shown to create an improved Sp1 binding site, leading to increased expression of the Mdm2 protein and thus attenuation of the p53 pathway and accelerated tumor formation in individuals carrying a germ-line p53 mutation [17-19]. A number of small studies revealed an inconsistent association between SNP309 and breast cancer risk (see overview in [1], and [20,21]). However, we have shown in a large pooled analyses of the Breast Cancer Association Consortium series that there is no general association of SNP309 with breast cancer, nor if stratified by estrogen receptor (ER) [1].

In two small studies no association between breast cancer survival and *MDM2 *SNP309 genotype alone was found [13,22]. However, the results of one of those studies suggested a differential effect of *MDM2 *SNP309 genotype by tumor p53 status (mutant p53 or aberrant protein expression) on breast cancer survival [22]. Though *MDM2 *SNP309 has been implicated to affect survival in other tumors (for example, [23]), as far as we know there are no other publications on breast cancer outcome and this polymorphism, except for a recent publication in *BRCA1/2 *carriers of Ashkenazi origin [24]. Our aim was to investigate the combined effects of *MDM2 *SNP309 and *TP53 *R72P polymorphisms and p53 protein expression on breast cancer survival.

## Materials and methods

### Clinico-pathologic data and genotyping

Breast cancer cases from four European studies within the Breast Cancer Association Consortium were included in this analysis (Table 1) [1,25]. Patients that were genotyped for *MDM2 *SNP309 and *TP53 *R72P from studies with follow-up data were included [1]. Patient selection criteria, participation rates and information on the collection of follow-up and clinical data are shown in Table 1. P53 protein expression data were available for two of the four studies (Table 1). Immunohistochemical staining of TMA slides was performed with a mouse monoclonal anti-human p53-antibody (DO-7, DAKO) (Table 1). Missing p53 data could be attributed to missing tumor blocks, loss of cores in the slicing or staining process or cores not containing enough tumor material. P53 protein expression scoring and *MDM2 *SNP309 and *TP53 *R72P genotyping were performed blinded to the survival status of the patients. Genotyping assays were performed by each group separately [1] (see Table 1 for assay description). Primer (and probe) sequences are available from the authors upon request. Methods and results in this paper are reported following the REMARK recommendations [26]. All studies were approved by the appropriate (Medical) Ethical Research Committees.

**Table 1:** Characteristics of the studies and genotyping assays

Contributing studies	Design	Description of case subjects and ascertainment (age range)	Participation rates	Follow-up	P53 IHC*	**Genotyping platform(s) **[38-40]
ABCS: Amsterdam Breast Cancer Study, The Netherlands [41]	Hospital-based consecutive cases	All operable breast cancer patients aged < 50 years diagnosed 1974-1994 in four Dutch hospitals (Amsterdam and Leiden) (23 to 50 years)	All patients with paraffin-embedded tissue blocks available (normal tissue) from the Pathology archives and successful DNA isolation (approximately 85%)	Active follow-up through the medical registries and general practitioners	By IHC staining of TMAs* as previously described [25]; p53 positive defined as > 10% of cells with positive nuclear staining.	Taqman
HABCS: Germany: Hannover Breast Cancer Study and bilateral breast cancer patients [42,43]	Hospital-based case-control studies	Case patients who received radiotherapy for breast cancer at Hannover Medical School between 1997 and 2003 (27 to 91 years)	Approximately 80% of case subjects contacted agreed to give a blood sample	Active follow-up at the Department of Radiation Oncology, Hannover Medical School	NA	Restriction enzyme-based assays
HEBCS: Helsinki Breast Cancer Study [10,44]	Hospital-based case-control study	Consecutive incident cases from the Department of Oncology, Helsinki University Central Hospital 1997-1998 (22 to 96 years)	79% of the case subjects	Active follow-up of the medical records until five years and annual linkage to the nation-wide Finnish Cancer Registry	By IHC staining of TMAs* as previously described [10] and data for 23 cases derived from the pathology reports; p53 positive defined as > 20% of cells with positive nuclear staining.	RFLP (*MDM2 *SNP309) Amplifluor(tm) fluorescent genotyping (Kbiosciences) (*TP53 *R72P)
SEARCH: Studies of Epidemiology and Risk Factors in Cancer Heredity, Cambridge, UK [45]	Population-based case-control study	Two groups of case patients (prevalent and incident) identified through East Anglian Cancer Registry: patients diagnosed before age 55 years in 1991 to 1996 and still alive when study started in 1996 and patients diagnosed before age 70 years since 1996 (25 to 65 years)	64% of eligible case subjects provided a blood sample	Combination of passive follow-up through national death registrations and active follow up every five years by the cancer registry	NA	Taqman

*IHC = immunohistochemistry; TMA = Tissue Micro Array; NA = not applicable (no p53 data available).

### Statistical analyses

Univariate analyses of survival were performed by calculating Kaplan-Meier survival curves and comparing subsets of patients using log-rank test. To explore the effects of several variables and their combined effects on survival, multivariate Cox's proportional hazards regression models were used (reported as Hazard Ratio (HR) with 95% confidence interval). Results are reported for one polymorphisms stratified by the other polymorphisms or p53 expression, adjusted for other covariates. Interaction terms were tested by Cox regression models including the main effects (2df each), interaction terms, for example, four interaction terms for both polymorphisms, and other covariates. Covariates included were prognostic factors for breast cancer survival, that is, age, stage, grade and ER and p53 protein expression. In order to run models including all patients, missing value categories were included for each separate variable with missing information. Polymorphisms were included as categorical variables (with the homozygous common allele group as reference), or as a continuous variable in the per-allele analyses. All pooled analyses were adjusted for study, that is, ABCS, HABCS, HEBCS, SEARCH, included as a categorical variable. Breast cancer-specific survival was defined as survival until death from breast cancer, with breast cancer being the underlying cause of death; death due to other causes was censored (these analyses included the ABCS and HEBCS studies, see Table 1). Overall survival was defined as survival until death of any cause. In all analyses, follow-up time was censored at 10 years. All statistical tests used were two-sided and *P *values < 0.05 were considered statistically significant. All analyses were performed using SPSS 15.0 (SPSS Inc, Chicago, IL, USA).

## Results

### Patient characteristics

Breast cancer patients with follow-up and *TP53 *R72P and *MDM2 *SNP309 genotypes from three hospital-based and one population-based study within the Breast Cancer Association Consortium were included for analysis (n = 3,749) (Table 1). Frequencies of *TP53 *R72P and *MDM2 *SNP309 and clinicopathologic characteristics of the breast cancer patients in the four studies are shown in Table 2. We have described and discussed earlier the small difference in *MDM2 *SNP309 allele frequencies between European populations [1] while difference in patient characteristics between studies can mostly be attributed to differences in patient selection criteria (Table 1). Mean follow-up was 7.7 years (SD 4). A small number patients (n = 26) were carriers of the homozygous rare variants for both polymorphisms (Table 3).

**Table 2:** Germ-line variants and clinicopathologic characteristics of breast cancer patients by study

		ABCS N = 1076		HABCS N = 152		HEBCS N = 599		SEARCH N = 1922		P value*
						
		N	%	N	%	N	%	N	%	
***MDM2 *SNP309**	**TT**	444	41.3	55	36.2	183	30.6	774	40.3	
	**GT**	487	45.3	73	48.0	311	51.9	913	47.5	
	**GG**	145	13.5	24	15.8	105	17.5	235	12.2	< 0.001

***TP53 *R72P**	**GG**	570	53.0	85	55.9	314	52.4	1052	54.7	
	**GC**	422	39.2	55	36.2	236	39.4	733	38.1	
	**CC**	84	7.8	12	7.9	49	8.2	137	7.1	0.9

**Stage**	**1**	341	31.9	83	69.2	205	36.9	861	52.4	
	**2**	581	54.4	36	30.0	295	53.1	713	43.4	
	**3**	146	13.7	1	0.8	56	10.1	69	4.2	< 0.001
	**Missing**	8		32		43		279		

**Differentiation grade**	**1**	338	35.8	9	8.7	138	24.6	368	25.4	
	**2**	317	33.6	55	53.4	243	43.2	647	44.7	
	**3**	288	30.5	39	37.9	181	32.2	431	29.8	< 0.001
	**Missing**	133		49		37		476		

**ER status tumor**	**Negative**	240	34.2	18	15.4	135	23.2	175	19.8	
	**Positive**	461	65.8	99	84.6	446	76.8	708	80.2	< 0.001
	**Missing**	375		35		18		1039		

**p53 status tumor**	**Negative**	473	70.4			335	76.7			
	**Positive**	199	29.6			102	23.3			0.02
	**Missing**	404		152		162		1922		

**Vital status patient**	**Alive**	694	64.5	129	84.9	462	77.1	1596	83.0	
	**Deceased, all**	382	35.5	23	15.1	137	22.9	326	17.0	< 0.001
	**Deceased, breast cancer**	337		20		105				

**Years of diagnosis**	**Range**	1974 to 1994		1997 to 2003		1997 to 1998		1991 to 1996		

**Age at diagnosis**	**Mean ± SD**	42.8	5.2	56.8	11.3	56.4	12.8	50.1	7.7	< 0.001

**Follow-up**	**Mean ± SD**	10.5	5.7	6.5	1.9	7.3	2.1	6.3	2.1	< 0.001

* *P *value of comparison of either categories of non-missing data among studies (by chi-square) or comparison of continuous data (by t-test).

**Table 3:** Frequencies of *TP53 *R72P and *MDM2 *SNP309 germ-line variants

*TP53 *R72P	GG		GC		CC	
			
	N	%	N	%	N	%
***MDM2 *SNP309 TT**	799	54.9	546	37.5	111	7.6
**GT**	940	52.7	699	39.2	145	8.1
**GG**	282	55.4	201	39.5	26	5.1

### Breast cancer survival by TP53 R72P, MDM2 SNP309 genotype, and p53 tumor status

Overall survival of patients did not differ by carriership of either germ-line variant, R72P or SNP309, alone in the pooled analyses (Table 4). Tumor p53 status was available for 1109 patients from the ABCS and HEBCS series (Table 1). In both series, the patients with p53-positive tumors showed poorer overall survival than the patients with p53-negative tumors (pooled HR 1.5 (1.2-1.9), *P *= 0.002; Table 4).

**Table 4:** HR estimates of overall survival* by *TP53 *R72P, *MDM2 *SNP309 and p53

*TP53 *R72P**	**HR**	**Lower and upper limit 95% CI**		***P *value**
			
**ABCS**				
**GC**	0.99	0.79	1.26	0.95
**CC**	0.72	0.45	1.16	0.17
**HABCS**				
**GC**	1.40	0.60	3.45	0.46
**CC**	2.54	0.69	9.34	0.16
**HEBCS**				
**GC**	1.17	0.82	1.67	0.40
**CC**	1.72	1.00	2.98	0.05
**SEARCH**				
**GC**	1.18	0.95	1.49	0.14
**CC**	0.93	0.59	1.48	0.77
**Pooled**^†^				
**GC**	1.11	0.96	1.28	0.18
**CC**	1.00	0.76	1.31	0.97

*MDM2 *SNP309**				
			
**ABCS**				
**TG**	0.93	0.73	1.18	0.54
**GG**	0.99	0.70	1.40	0.97
**HABCS**				
**TG**	0.60	0.25	1.46	0.26
**GG**	0.36	0.08	1.65	0.19
**HEBCS**				
**TG**	0.76	0.53	1.11	0.16
**GG**	0.91	0.56	1.47	0.69
**SEARCH**				
**TG**	1.03	0.82	1.31	0.78
**GG**	1.43	1.03	1.97	0.03
**Pooled**^†^				
**TG**	0.93	0.80	1.08	0.34
**GG**	1.11	0.90	1.37	0.31

p53 status tumor **				
			
**ABCS**				
**p53 positive**	1.31	0.96	1.80	0.09
**HEBCS**				
**p53 positive**	1.93	1.27	2.94	0.002
**Pooled**^†^				
**p53 positive**	1.50	1.16	1.93	0.002
**Breast cancer-specific survival** **Pooled**^†^				
**p53 positive**	1.57	1.20	2.05	0.001

*Overall survival including all studies unless otherwise specified; ** HRs of heterozygous and homozygous rare allele groups have been calculated by comparison to the reference categories of common alleles: *TP53 *R72P = GG; *MDM2 *SNP309 = TT; p53 = negative tumors; ^†^Pooled analyses have been adjusted for study.

### Differential effect of TP53 R72P on breast cancer survival stratified for p53 tumor status

**Figure 1:**
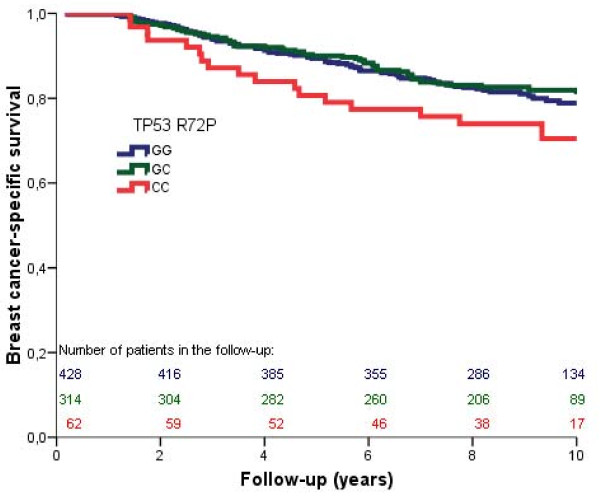
Cumulative breast cancer-specific survival (Kaplan Meier) of breast cancer patients with p53 negative tumors stratified by *TP53 *R72P. Survival in the *TP53 *CC group was worse compared to that in the GC and GG group combined (80% versus 71%, *P *= 0.07).

In the patient group with p53-negative tumors, the actuarial breast cancer-specific survival for the patients carrying the *TP53 *CC genotype (Pro/Pro) was worse, though not statistically significantly, at 10 years of follow-up as compared to those carrying *TP53 *GG/GC (Arg/Arg; Arg/Pro) (71% versus 80% *P *= 0.07; Figure 1). The interaction terms between p53 expression and *TP53 *R72P were not significant in a multivariate Cox regression analysis, but considering the difference seen in the actuarial curves we still considered it useful to perform Cox analyses stratified for p53 expression. Patients with the *TP53 *CC genotype had worse breast-cancer specific survival (HR adjusted for study, age, stage, grade and ER: 1.79 (1.05 to 3.05, *P *= 0.03) (Table 5). Results for overall survival were in line with those of breast-cancer specific survival, but did not reach statistical significance (*P *= 0.06, Table 5).

**Table 5:** HR estimates of overall and breast cancer-specific survival by *TP53 *R72P, in p53 negative and positive tumors (multivariate models)

***TP53 *R72P**	**HR**	**Lower and upper limit 95% CI**		***P *value**
			
**Overall survival**				
**p53 negative tumors**				
**GC**	1.11	0.80	1.52	0.54
**CC**	1.63	0.97	2.74	0.06
**p53 positive tumors**				
**GC**	0.82	0.53	1.27	0.37
**CC**	0.86	0.34	2.18	0.75

**Breast cancer-specific survival**				
**p53 negative tumors**				
**GC**	0.97	0.68	1.37	0.85
**CC**	1.79	1.05	3.05	0.03
**p53 positive tumors**				
**GC**	0.90	0.57	1.42	0.65
**CC**	1.00	0.39	2.55	1.00

***MDM2 SNP309***	**HR**	**Lower and upper limit 95% CI**		***P *value**
			
**Overall survival**				
**p53 negative tumors**				
**TG**	0.84	0.60	1.17	0.30
**GG**	1.28	0.85	1.94	0.24
**p53 positive tumors**				
**TG**	0.78	0.49	1.24	0.30
**GG**	0.78	0.41	1.48	0.45

**Breast cancer-specific survival**				
**p53 negative tumors**				
**TG**	0.92	0.64	1.31	0.63
**GG**	1.41	0.90	2.19	0.13
**p53 positive tumors**				
**TG**	0.76	0.47	1.23	0.27
**GG**	0.69	0.35	1.39	0.30

Pooled analyses for studies with p53 information (ABCS and HEBCS); HRs of heterozygous and homozygous rare allele groups have been calculated by comparison to the reference categories of the homozygous common allele: *TP53 *R72P = GG, and *MDM2 *SNP309 = TT; analyses have been adjusted for study, age, stage, grade and ER.

Within the patient group with p53-positive tumors, breast cancer-specific survival stratified by *TP53 *R72P seemed to show inconsistent results between studies though none were significant, that is, per allele HR (adjusted for age, stage, grade and ER) in the ABCS study was 0.74 (0.45 to 1.22) and in the HEBCS study 1.46 (0.79 to 2.69). The pooled HR (adjusted for study, age, stage, grade and ER) was 0.82 (0.53 to 1.27) for heterozygous and 0.86 (0.34 to 2.18) for homozygous C-allele carriers (Table 5). There was no evidence for a differential effect of *MDM2 *SNP309 by p53 tumor status on survival (Table 5).

### Combined effects of TP53 R72P and MDM2 SNP309 on breast cancer survival

**Figure 2:**
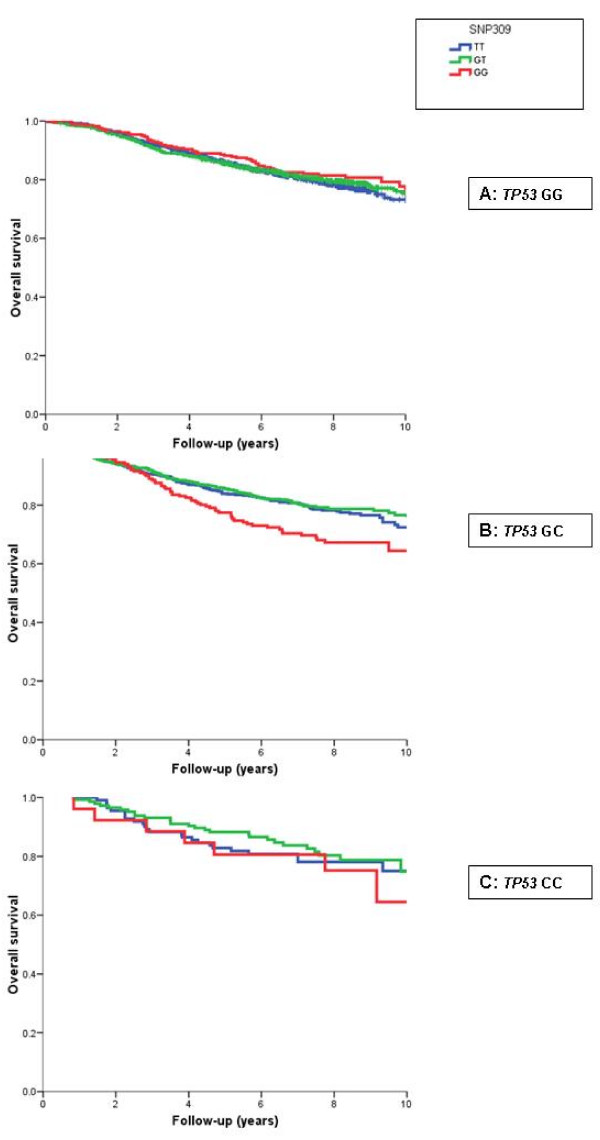
Cumulative overall survival of breast cancer patients by *MDM2 *SNP309 and *TP53 *R72P genotypes. Each figure shows Kaplan Meier survival curves of *MDM2 *SNP309 genotypes within one group of *TP53 *R72P genotype. **(a) ***TP53 *GG genotype (ns); **(b) ***TP53 *GC genotype (*P *= 0.006); **(c) ***TP53 *CC genotype (ns). The numbers at start of follow-up were: Figure A: TT n = 798, TG n = 939, GG n = 281; B: TT n = 545, TG n = 698, GG n = 200; C: TT n = 110, TG n = 144, GG n = 25. Within the TP53 C-allele carriers (Figure A and B combined), *MDM2 *GG carriers had significantly worse survival compared to TT/TG carriers combined: 64% versus 75%, *P *= 0.001.

*MDM2 *SNP309 showed a differential actuarial overall survival stratified by *TP53 *R72P in the pooled analyses (n = 3,749), that is, homozygous carriers of the G-allele in *MDM2 *had worse survival within the group of *TP53 *GC carriers (GG: 65% versus GT: 72% and TT: 76%, *P *= 0.006; Figure 2). The same trend was visible in the *TP53 *homozygous CC group (n = 26 GG/CC), but this was not statistically significant. Within the *TP53 *C-allele carriers combined, *MDM2 *GG carriers had significantly worse survival compared to TT/TG carriers: 64% versus 75%, *P *= 0.001. In multivariate analyses (adjusting for study, age, stage, grade and ER) the interaction term for *TP53 *GC and *MDM2 *GG was significant (*P *= 0.028), also if additional interaction terms for *TP53 *R72P and p53 expression were included (*P *= 0.027). The multivariate models (adjusting for study, age, stage, grade and ER) stratified for *TP53 *R72P (analogue to Figure 2) showed that *MDM2 *GG carriers had significantly worse survival compared with *MDM2 *TT carriers only within the *TP53 *C-allele carriers; more specifically, within *TP53 *CG carriers: HR 1.43 (1.05 to 1.96), *P *= 0.02; within *TP53 *CC carriers HR 1.39 (0.56 to 3.48), *P *= 0.48 (Table 6); within *TP53 *CG and CC carriers combined: HR (adjusted for study, age, stage, grade and ER) 1.46 (1.09 to 1.96), *P *= 0.01.

**Table 6:** HR estimates of multivariate analyses for *MDM2 *SNP309 stratified by *TP53 *R72P

	**HR**	**Lower and upper limit 95% CI**		***P *value**	**HR**	**Lower and upper limit 95% CI**		***P *value**	**HR**	**Lower and upper limit 95% CI**		***P *value**
									
	***TP53 *R72P: GG**				***TP53 *R72P: GC**				***TP53 *R72P: CC**			
			
***MDM2 *SNP309 TT**	1.0 (Ref)				1.0 (Ref)				1.0 (Ref)			
**TG**	.90	.73	1.11	.33	.94	.73	1.20	.61	.85	.47	1.55	.60
**GG**	.85	.63	1.16	.31	1.43	1.05	1.96	.02	1.39	.56	3.48	.48

**p53 negative**	1.0 (Ref)				1.0 (Ref)				1.0 (Ref)			
**positive**	1.37	.96	1.96	.08	.89	.58	1.35	.57	.76	.25	2.30	.63
**missing**	1.63	1.16	2.28	.005	1.66	1.13	2.44	.009	.53	.21	1.34	.18

**Stage 1**	1.0 (Ref)				1.0 (Ref)				1.0 (Ref)			
**2**	2.51	1.90	3.33	< .001	2.55	1.88	3.46	< .001	1.48	.73	2.97	.28
**3**	7.10	5.05	9.97	< .001	7.56	5.18	11.02	< .001	5.49	2.17	13.87	< .001
**missing**	3.69	2.42	5.63	< .001	2.55	1.54	4.21	< .001	1.60	.46	5.57	.46

**Grade 1**	1.0 (Ref)				1.0 (Ref)				1.0 (Ref)			
**2**	1.80	1.27	2.54	.001	1.12	.77	1.62	.55	3.28	1.29	8.35	.01
**3**	2.63	1.84	3.76	< .001	2.39	1.65	3.46	< .001	4.08	1.55	10.79	.005
**missing**	1.84	1.21	2.78	.004	1.391	.87	2.22	.17	2.11	.64	6.92	.22

**ER negative**	1.0 (Ref)				1.0 (Ref)				1.0 (Ref)			
**positive**	.63	.48	.84	.002	.61	.44	.83	.002	.59	.27	1.26	.17
**missing**	.65	.46	.91	.01	.58	.40	.84	.003	.90	.36	2.25	.82

**Age**	1.02	1.01	1.03	.001	1.00	.99	1.02	.54	1.01	.98	1.04	.70

Models have also been adjusted for study.

## Discussion

In the survival analyses including 3,749 breast cancer patients from Finland, The Netherlands, Germany and United Kingdom, we showed combined effects of two germ-line polymorphisms, *TP53 *R72P, *MDM2 *SNP309, and p53 tumor expression (by immunohistochemistry). Firstly, we confirmed our earlier observation in Finnish patients [10] that *TP53 *R72P homozygous carriership predicts a worse survival in patients with p53-negative tumors, also when adjusted for clinical prognostic variables. Thus, in the absence of inactivating p53 mutations in the tumor, the 72P variant form of p53 protein may have a compromising effect on the p53 apoptotic function, leading to reduced survival of the patients. Similarly, a study of 414 Chinese breast cancer patients reported that the 72P homozygous (CC) genotype was associated with both poorer five-year overall survival (five to eight percentile difference, *P *= 0.04) and poorer disease-free survival among the patients with a wild-type p53 in their tumors (n = 346) [16]. In line with other studies published we did not observe an effect of carriership of R72P alone on survival of patients [12-16].

No significant difference in survival by *TP53 *R72P carriership was observed among the patients with p53-positive tumors, who showed a worse survival overall compared to p53-negative tumors. In the pooled analysis, CC homozygote patients with p53-positive tumors even tended to have a better survival. In the study by Xu et al. in Chinese breast cancer patients [16], the CC homozygote patients also had non-significant better survival than the GG homozygotes and heterozygotes within the group of patients with p53-mutated tumors.

The finding of CC homozygote (72P) carriers having poorer survival is consistent with the R72 variant of wild-type p53 being a more potent inducer of apoptosis than the wild-type 72P variant. It has been suggested that R72 homozygotes may respond more favorably to radiation or chemotherapy [27]. Response rate after chemo-radiotherapy of advanced squamous cell carcinomas of head and neck and survival was higher in patients with the R72 allele compared to those with the 72P allele [28]. These favorable effects of the R72 allele may, however, be reversed by a somatic p53 mutation on this allele, as has been reported in squamous cell carcinomas of head and neck [29,30]. In line with this, retention of the R72 allele with loss of the 72P allele in the tumor tissue has been associated with reduced survival in heterozygous breast cancer patients [31].

Carriership of *MDM2 *SNP309 alone did not affect survival of patients in our study and two other, smaller studies [13,22]. However, we found an 11 percentile survival difference for homozygous *MDM2 *G-allele carriers within the group of *TP53 *C-allele (72P) carriers. Biologically this seems plausible considering the reduced apoptotic function of the *TP53 *Pro-variant [6,7] and the attenuation of the p53 pathway by mdm2, the production of which is increased by the SNP309 G-variant [17]. In addition, the interaction of both polymorphisms remained statistically significant in multivariate models adjusting for clinical prognostic factors.

We did not observe evidence for a combined effect of SNP309 and p53 tumor expression (as shown here by results of SNP309 stratified by p53 status in Table 5, but obviously p53 did also not have a differential effect on survival stratified by SNP309). This is in contrast to a previous, smaller study (n = 248) in the American population, which suggested that tumor p53 status was associated with breast cancer survival only among patients homozygous for the *MDM2 *SNP309 T-allele and not among carriers of the variant G-allele [22]. Though our study is one of the largest published studies on combined effects of the germline genetic variation and tumor somatic events, the numbers are still small for looking at such modifying effects on survival.

Many studies have confirmed that mutated p53 is a prognostic factor in breast cancer. The risk of dying of breast cancer for patients with a p53 mutation in their tumor has been estimated to be two to five-fold compared to patients with wild-type p53 tumors [32,33]. Positive immunostaining for p53 is in general considered to indicate somatic p53 mutation and an impaired p53 pathway, though the correlation with *TP53 *mutations is incomplete [34,35]. The accumulation of p53 in the tumors detected by immunohistochemistry was a prognostic marker of poorer survival in both our series with p53 immunohistochemistry data available (the HEBCS and ABCS series). This effect was somewhat stronger in the HEBCS series, which may be explained by the more stringent cut-off used (20% positive tumor cells compared to 10% in the ABCS series).

## Conclusions

We have shown here that *TP53 *R72P may have additional prognostic value especially among patients with p53-negative tumors. However, the effect of p53 on outcome may be influenced by adjuvant systemic therapy (for example, [31,36], reviewed in Bertheau [37]) and larger studies will be needed to address this question. Our study is one of the few that have shown an interaction of germ-line variants, that is, *TP53 *R72P and *MDM2 *SNP309, in breast cancer survival. The results, showing a statistically significant interaction of the p53 Pro-variant and the GG genotype of *MDM2 *SNP309, are in line with our a priori biologically-supported hypothesis, which is, the role of enhanced DNA repair function of the Pro-variant, combined with increased expression of the Mdm2 protein, and thus overall attenuation of the p53 pathway in the tumor cells. These results suggest that even subtle differences in p53 apoptotic function caused by synergistic polymorphisms may affect patient's survival, possibly by modifying treatment response. Altogether, our findings are in line with biological evidence in literature, and in the future, may have also clinical significance for models of breast cancer prognosis or treatment. However, because this is the first report on the combined effect of *TP53 *R72P and *MDM2 *SNP309 on breast cancer survival and we cannot exclude a chance finding, other studies to confirm this will be necessary. Larger studies will be needed also to investigate the effect of specific treatment modalities on the survival by *TP53 *R72P and *MDM2 *SNP309.

## Abbreviations

ABCS: Amsterdam Breast Cancer Study; ER: estrogen receptor; HABCS: Hannover Breast Cancer Study; HEBCS: Helsinki Breast Cancer Study; HR: hazard ratio; SD: standard deviation; SEARCH: Studies of Epidemiology and Risk Factors in Cancer Heredity; TMA: tissue micro array.

## Competing interests

The authors declare that they have no competing interests.

## Authors' contributions

MKS, TD and HN took final responsibility for the decision to submit the paper for publication; all other authors read and approved the manuscript. MKS, JT, FEVL, LJVTV, PDPP, DFE, TD, CB, HN were responsible for the study design. MKS, JT, AB, MS, MH, TD, SAR, RF were responsible for data acquisition and collection. MKS and JT did the data analyses. Data interpretation was carried out by MKS, JT, AB, RF, CB, and HN. MKS, AB, TD, CB, and HN wrote the paper. All authors read and approved the manuscript.
